# Drought‐response strategies of savanna herbivores

**DOI:** 10.1002/ece3.5270

**Published:** 2019-05-22

**Authors:** Joel O. Abraham, Gareth P. Hempson, A. Carla Staver

**Affiliations:** ^1^ Department of Ecology and Evolutionary Biology Yale University New Haven Connecticut; ^2^ South African Environmental Observation Network (SAEON), Ndlovu Node Pretoria South Africa; ^3^ Centre for African Ecology, School of Animal, Plant and Environmental Sciences University of the Witwatersrand Wits South Africa

**Keywords:** diet switching, drought, drought refugia, herbivory, migration, savanna

## Abstract

1. Climate models predict increases in drought frequency and severity worldwide, with potential impacts on diverse systems, including African savannas. These droughts pose a concern for the conservation of savanna mammal communities, such that understanding how different species respond to drought is vital.
2. Because grass decreases so consistently during droughts, we predict that grass‐dependent species (grazers and mixed feeders) will respond strongly to drought, whether by changing diets, seeking drought refugia, or suffering mortality.
3. A recent severe but heterogeneous drought in Kruger National Park, South Africa, afforded a rare opportunity to test these hypotheses in situ—crucial, given the central role of landscape‐scale movement as a potential herbivore strategy. We used herbivore dung as a proxy, integrating spatial distributions (dung counts) with diet composition (carbon isotope analysis of dung).
4. As predicted, browsers showed little response to drought. However, mixed feeders switched their diets to incorporate more C3 trees/forbs, but did not move. Meanwhile, grazers and megaherbivores instead moved toward drought refugia.
5. *Synthesis and applications*: The responses we observed by savanna herbivores are largely amplifications of typical dry season strategies and reflect constraints imposed by body size and feeding ecology. Grazers may be at particular risk from increased drought frequency and spatial extent if drought refugia become decreasingly available. Conservation strategies should recognize these constraints and work to facilitate the diverse responses of herbivores to drought.

## 1 INTRODUCTION

African savannas are home to the world's most abundant and diverse communities of large mammalian herbivores (DuToit & Cumming, 1999). This diversity of herbivores is reflected in the functional diversity they display, including dramatic diet (Codron et al., 2007) and body size (Hempson, Archibald, & Bond, 2015) variation that contributes to their coexistence (Kartzinel et al., 2015) and shapes their impacts on savanna vegetation. However, these herbivores are threatened by various aspects of global change (Ripple et al., 2015), including probable increases in the incidence of drought (Knapp et al., 2008). Droughts often induce widespread and catastrophic mortality among savanna herbivores (Young, 1994), and as such, effective conservation of these herbivores will depend on understanding the strategies they use to mitigate drought‐induced mortality. Droughts are, however, infrequent and unpredictable, and it is impossible to experimentally evaluate landscape‐scale animal responses to drought. Thus, rigorous explorations of herbivore responses to drought are limited.

Of course, savannas are variable systems even under normal conditions, often characterized by pronounced dry and wet seasons (Staver, Archibald, & Levin, 2011). The dry season causes rapid reductions in grass forage quality as grasses become dormant, whereas tree species tend to retain their leaves and are much more variable in their responses to rainfall seasonality (Ryan, Williams, Grace, Woollen, & Lehmann, 2016). Because grass forage quality declines predictably during the dry season, whereas trees may not, grazing herbivores especially rely on seasonal strategies for mitigating scarcity. They utilize two main strategies: diet flexibility (Codron et al., 2007) and migration (Fryxell & Sinclair, 1988). In the case of diet flexibility, mixed feeders, which primarily graze in the wet season, switch to browsing in the dry season (alongside exclusive browsers) as grass nutrition and biomass decline (Codron et al., 2007). In the case of migration, seasonal shifts are spatial instead of compositional, and seasonal migration of grazers is (Frank, McNaughton, & Tracy, 1998) or was (Harris, Thirgood, Hopcraft, Cromsigt, & Berger, 2009) common, both in Africa and beyond.

Dry season strategies may shape how herbivores respond to longer‐term droughts, too, but predictions diverge as to exactly how different herbivores should respond to drought. Plant responses to drought parallel normal dry season responses; droughts can have extended interannual legacy effects on grass diversity and biomass (Sala, Gherardi, Reichmann, Jobbagy, & Peters, 2012), but tree responses to drought are much less consistent (Augustine, 2010 Case et al., in revision; Sankaran, in press; [Link]; Walker, Emslie, Owen‐Smith, & Scholes, 1987), with some tree species largely unaffected. Thus, seasonal diet and movement strategies might also determine herbivore drought responses, particularly if seasonal herbivore behaviors are constrained by their morphologies (Fryxell & Sinclair, 1988; Gordon, 2003). In this case, perhaps mixed feeders cope with drought by eating more browse, while grazers move to areas less severely affected by drought when they can (Staver, Wigley‐Coetsee, & Botha, 2019) or experience larger population declines (Augustine, 2010). An alternative possibility is that droughts may qualitatively change herbivore behaviors, such that seasonal strategies do not predict drought mitigation strategies. Certainly, a growing literature relying on evidence from DNA metabarcoding suggests that all herbivore diets, and not just classic mixed feeders, may be more variable than the “feeding guild” paradigm suggests (Kartzinel et al., 2015). Grazers and mixed feeders alike may therefore increase their dietary breadth to take advantage of leafy trees or drought‐specialist forbs (O'Connor, 1995), and all herbivores may also move in the landscape to take advantage of forage reservoirs that are little‐used during normal conditions (Riginos, 2015).

Body size may partly determine how flexible—or indeed how necessary—herbivore strategies may be, via restrictions on movement (Augustine, 2010), predation avoidance (Hopcraft, Olff, & Sinclair, 2010), and metabolism (Olff, Ritchie, & Prins, 2002). Firstly, body size can dictate herbivore mobility: Larger herbivores are less impeded by landscape barriers and can make more efficient use of their greater fat reserves (Damuth, 1981; Wittemyer, Getz, Vollrath, & Douglas‐Hamilton, 2007), such that they are more able to travel long distances (Hein, Hou, & Gillooly, 2012; Owen‐Smith, 1988). Secondly, body size clearly impacts rates of predation (Hopcraft et al., 2010; Sinclair, Mduma, & Brashares, 2003), such that smaller bodied herbivores may be more constrained in their landscape use than larger ones (Riginos, 2015). And finally, diet quality–quantity trade‐offs likely play a role (Olff et al., 2002)—the digestive efficiency of larger herbivores allows them to tolerate plants with lower nutrient content, but megaherbivores require food in bulk, perhaps forcing large herbivores to feed more generally and range more widely during drought to fulfill these requirements (Riginos, 2015). These factors together may mean that larger herbivores can and need to change their diet and/or landscape use during drought.

Overall, we predict that diet flexibility and migration represent important potential strategies that herbivores might employ in response to drought stress. Here, we examine (a) whether herbivores of different body sizes and feeding guilds demonstrate distinct behaviors to respond to drought, and (b) whether these strategic responses predict overall herbivore mortality during drought. To understand the behavioral responses of savanna herbivores to drought, we compare herbivore landscape use and diet composition in Kruger National Park in South Africa during and after a severe but heterogeneous drought. Finally, we evaluate herbivore success during drought, via an analysis of published estimates of drought‐driven herbivore mortality (Young, 1994). The Kruger drought was the first in 20 years in this system and, with a duration of at least 2 years, is one of the most severe on record. As such, this study represents a rare opportunity to study a phenomenon that is impossible to replicate experimentally. We expect these results to provide critical missing information for the conservation of mammalian herbivores in the face of increasing drought severity and frequency resulting from global change.

## 2 MATERIALS AND METHODS

### 2.1 Study site

Kruger National Park encompasses ~20,000 km^2^ (22°20′ to 25°30′S; 31°10′ to 32°00′E) in northeastern South Africa. The park spans a broad rainfall gradient, from <400 mm mean annual rainfall (MAR) in the north to >750 mm in the south (Staver, Botha, & Hedin, 2017). Rainfall is seasonal, falling mainly during summer, between October and March (Venter, Scholes, & Eckhardt, 2003).

**Figure 1:**
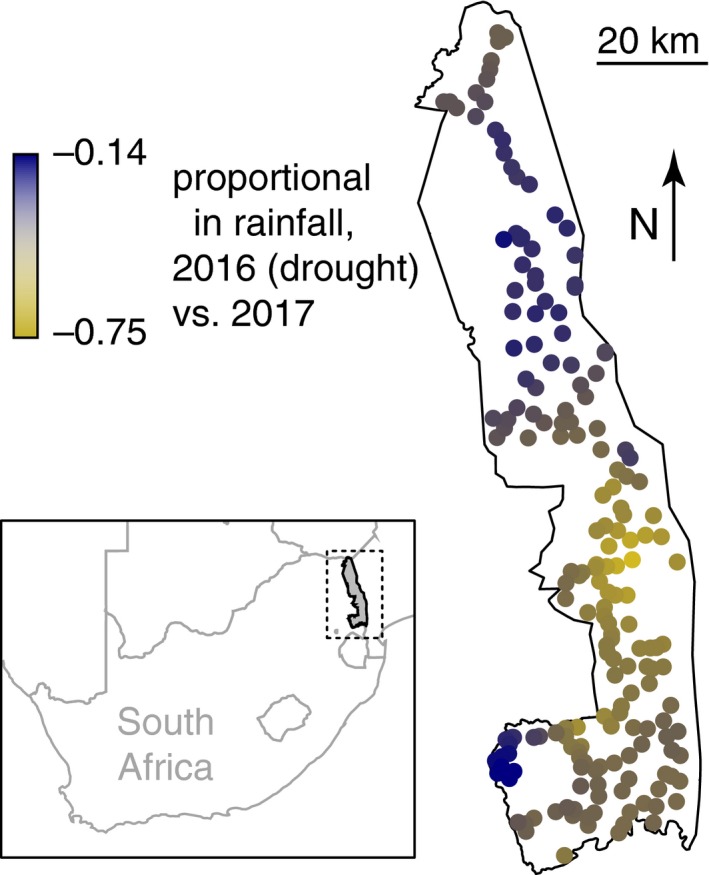
Proportional change in rainfall from 2016 (drought) compared with 2017 (postdrought) at dung‐count sites in Kruger National Park. Rainfall anomalies were extracted from interpolations across 22 weather stations throughout Kruger

The 2014–2016 drought was one of the most severe droughts on record in the region. However, Kruger spanned areas where the drought was severe (in the south) and less severe (in the north) (Figure 1; see also Staver et al., 2019). Local drought severity was calculated as a proportional change in rainfall from 2016 (drought) to 2017 (postdrought). We used 2016 and 2017 rainfall data drawn from a dataset continuously collected at 22 sites throughout Kruger and interpolated via inverse distance weighting to produce rainfall distribution maps across Kruger. We use 2017 rainfall data as a proxy for “normal” year rainfall to temporally match our herbivore sampling, which was likewise conducted in 2017 to represent “normal” conditions (see below).

Kruger is home to a full complement of native savanna herbivores, with total mean herbivore biomass ca. 6,080 kg/km^2^ (Staver et al., 2017). Impala (*Aepyceros melampus*) are by far the most numerous herbivore in Kruger (6.4 km^−2^ or 380 kg/km^2^), though elephants (*Loxodonta africana*) contribute the most biomass overall (0.7 km^−2^ or 1,900 kg/km^2^). Impala and elephants are mixed feeders (Codron et al., 2007, 2006). Meanwhile, browsers range from small herbivores, such as duiker (*Sylvicapra grimmia*) and steenbok (*Raphicerus campestris*), to larger herbivores, including kudu (*Tragelaphus strepsiceros*), giraffe (*Giraffa camelopardalis*), and the critically endangered black rhino (*Diceros bicornis*). Common grazers are warthog (*Phacochoerus africanus*), zebra (*Equus burchelli*), buffalo (*Syncerus caffer*), and white rhino (*Ceratotherium simum*).

Body mass estimates from Hempson et al. (2015) were used for Kruger herbivores (see Table 1). Herbivores with body masses of ca. 1,000 kg or larger are here considered to be megaherbivores (elephants, rhinos, and giraffes; Hopcraft et al., 2010), herbivores between 100 and 1,000 kg to be medium herbivores, and herbivores <100 kg to be small herbivores.

**Table 1:** Comparison of %C4 intake among Kruger National Park herbivores between a drought and postdrought year

Common name	Scientific name	Body mass (kg)^a^	%C4 in diet during drought (2016)			%C4 in diet postdrought (2017)				Baseline %C4 in diet^b^		
			*n*	Mean	*SD*	*n*	Mean	*SD*	*p* c	*n*	Mean	*SD*
Browsers												
Steenbok	*Raphicerus campestris*	11.2	9	2.6	2.5	18	0.8	1.8	1	17	6	15
Giraffe	*Giraffa camelopardalis*	1,117.5	4	5.9	3.6	16	1.5	2.7	1	99	5	5
Kudu	*Tragelaphus strepsiceros*	202.3	19	5.2	3.9	37	2.5	5.0	1	75	4	4
Duiker	*Sylvicapra grimmia*	16.9	9	12.0	8.1	18	5.8	12.5	1	8	5	5
Mixed feeders												
Elephant	*Loxodonta africana*	4,101.8	86	15.8	16.4	81	29.9	13.6	<0.001	946	41.2	18.0
Impala	*Aepyceros melampus*	49.1	73	33.3	13.5	79	49.1	21.3	<0.001	325	53	21
Grazers												
Waterbuck	*Kobus ellipsiprymnus*	211.8	6	74.1	19.9	6	78.5	24.7	1	47	90	6
Buffalo	*Syncerus caffer*	486.3	15	70.4	21.8	10	86.3	11.1	0.43	176	89	6
Wildebeest	*Connochaetes taurinus*	220.1	6	82.5	6.7	16	90.8	6.7	1	85	92	6
Zebra	*Equus burchelli*	280.4	20	89.0	5.1	18	91.1	9.9	1	81	94	5
Warthog	*Phacochoerus africanus*	75.9	5	95.0	5.1	7	98.2	3.3	1	32	92	7

Note

*n*, number of samples; *p*, *p*‐value; *SD*, ±1 standard deviation.

a Herbivore body mass data are from Hempson et al. (2015).

b Baseline data from Codron et al. (2007) correspond to a nondrought period and are included for the sake of comparison.

c *p‐*values correspond to Tukey's HSD.

### 2.2 Herbivore landscape use surveys

Though Kruger conducts uniquely comprehensive monitoring, spatially explicit data on herbivore populations are publically available for only elephant and buffalo. Therefore, to evaluate the fine‐scale distribution of herbivores across Kruger, we estimated herbivore landscape use via dung counts at 177 readily accessible veld condition assessment (VCA; for further description, see Staver et al., 2017) sites in May through July of both 2016 (drought) and 2017 (postdrought). Dung counts provide a detailed and spatially explicit proxy for herbivore landscape use intensity, though there is no guarantee that this equates to foraging intensity (Cromsigt, Rensburg, Etienne, & Olff, 2008).

Dung counts were performed along five 50‐m transects spaced 15 m apart within an established 50 m × 60 m grid at each site. All dung within two meters on either side of these transects was tallied by species. Dung was identified using Stuart and Stuart (2015), which provides species‐specific metrics for identifying dung, and identifications were verified by experienced field rangers. When dung piles overlapped (e.g., in middens and latrines), the number of piles was estimated from the quantity of dung per standard pile. This methodology resulted in species‐specific estimates of herbivore landscape use throughout Kruger.

### 2.3 Herbivore diet evaluation

In tropical savannas, stable carbon isotopes can be used to evaluate diet composition, because the isotopic composition of herbivore dung reliably reflects the relative proportions of C4 grasses versus C3‐photosynthesizing woody plants and forbs consumed (Codron et al., 2007). C3 and C4 photosynthetic pathways fractionate isotopes of carbon in CO_2_ differently, resulting in distinct δ^13^C signatures that are sufficient to overwhelm small changes in δ^13^C due to changes in plant water status or water‐use efficiency (Ehleringer, Hall, & Farquhar, 1993; Farquhar, Ehleringer, & Hubrick, 1989). Thus, it is possible to determine the relative amounts of grass and nongrass consumed by herbivores via carbon isotope analysis of their dung and a dual‐endpoint mixing model (Codron et al., 2007), though it is only possible to determine plant functional composition, not species identity or within‐functional‐type dietary breadth, via this method.

Herbivore dung was collected throughout Kruger primarily from VCA sites, in May through July of both 2016 (drought) and 2017 (postdrought). Fresh dung, visibly damp or wet, was opportunistically collected for isotope analysis from a diverse assemblage of herbivores representing an assortment of grazers, browsers, and mixed feeders. For less common taxa and for elephants, additional samples were collected along park roads. Some herbivores present in Kruger—hippos and rhinos, specifically—could not be included, as fresh dung was encountered infrequently. In total, 252 samples were collected in 2016 and 306 in 2017, representing 11 species in each year (see Table 1 for species‐specific sample numbers by year).

Samples were oven‐dried at 60°C for at least 72 hr before further processing. Dung was then ground using a mortar and pestle and/or ball mill. Ground samples were analyzed at the Yale Analytical and Stable Isotope Center (YASIC; detection limit c. 0.001% by weight) in December 2017 using a Costech ECS 4010 Elemental Combustion System (Costech Analytical Technologies) interfaced with a Thermo Delta Plus Advantage isotope ratio mass spectrometer (Thermo). ^13^C/^12^C ratios are expressed in the delta (δ) notation in parts per mil (‰) relative to the Vienna PeeDee Belemnite (VPDB) standard. Standard deviations of repeated measurements of laboratory standards were <0.1%.

All 2016 elephant dung samples had been run in December 2016 at the University of California, Davis, Stable Isotope Facility on a PDZ Europa ANCA‐GSL elemental analyzer interfaced to a PDZ Europa 20‐20 isotope ratio mass spectrometer (Sercon Ltd.). Twenty of these elephant dung samples were rerun at YASIC, and pairwise comparisons between the two facilities revealed that samples from UC Davis were consistently 0.03‰ greater, so isotope ratios for the samples analyzed only at UC Davis were corrected accordingly.

Fecal δ^13^C values were then converted into estimates of C4 grass intake via the equation:

$$\%C4_{\text{diet}}=\left(\delta^{13}C_{C3\;\text{- plants}}+\Delta \delta^{13}C-\delta^{13}C_{\text{feces}}\right)/\left(\delta^{13}C_{C3\;\text{- plants}}-\delta^{13}C_{C4\;\text{- plants}}\right)$$

where Δδ^13^C is the magnitude of discrimination against δ^13^C between consumer tissue and source endpoints (assumed here to be −0.9‰ for feces, Codron et al., 2007). Regionally specific baseline data for both C3 and C4 plants in Kruger were used to convert isotope values to diet composition: δ^13^C values were −27.35‰ and −13.20‰ for C3 and C4 plants, respectively (Codron et al., 2005). This approach facilitates dung δ^13^C values comparable to data from other similar studies (Codron et al., 2007).

### 2.4 Herbivore mortality estimates

Kruger herbivore population estimates during drought are not considered, even by park management, to be particularly robust without extensive statistical adjustment, such that direct examination of herbivore mortality during this drought is not possible here. Drought‐induced herbivore mortality estimates were therefore used from a published synthesis (Young, 1994). This synthesis catalogues published records of population declines (peak‐to‐trough population changes) that occurred between population surveys. Although this synthesis is somewhat out of date, it does span all previous droughts in the study region (the previous drought occurred in 1992) and is therefore effectively thorough. We included data for all species overlapping with this current work, yielding data for a subset (not full set) of the species included in other parts of this study (see Figure 4). Note also that the original synthesis only included events in which >25% of the population was lost, such that these estimates are biased toward large mortality events and against more resilient populations.

### 2.5 Data analysis

**Figure 2:**
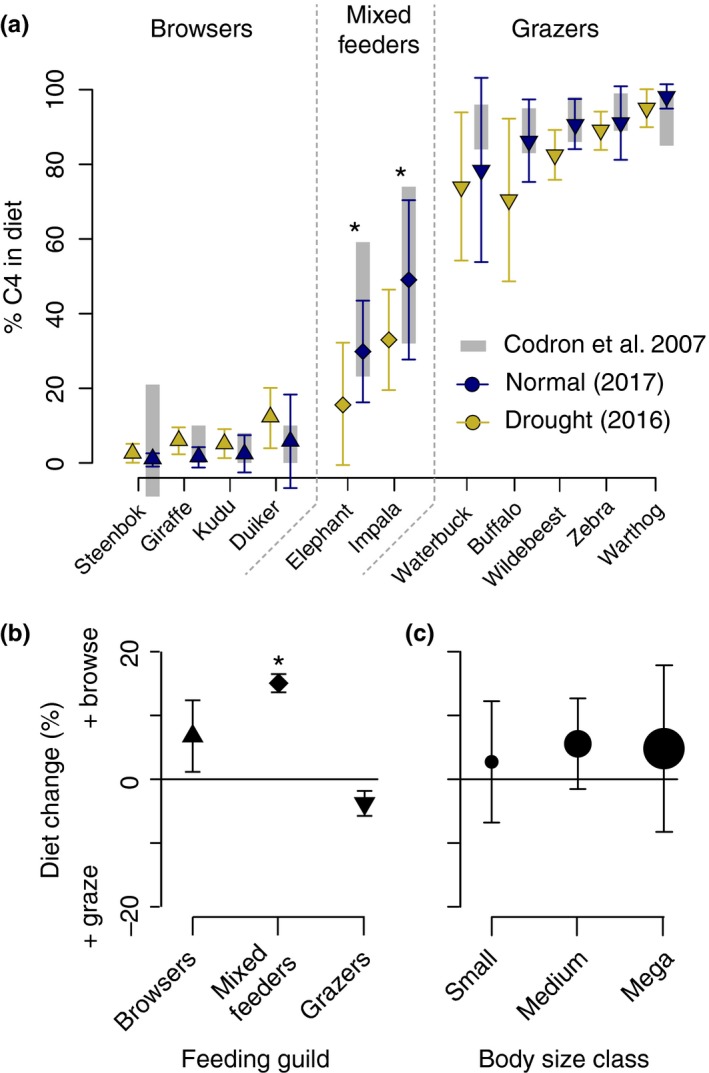
Diet composition, estimated from δ^13^C values during and after drought, by species (a), feeding guild (b), and body size class (c). Percentage C4 grasses in the diet (mean ± *SD*) was compared for drought versus postdrought for each species (a) or aggregated by feeding guild (b) and body size class (c). Values outside 0% to 100% arise when δ^13^C values fall beyond the range of end‐member values for C3 (−27.35‰) and C4 (−13.20‰) plants. Asterisks (*) denote significant difference (*p* < 0.0001, Tukey's HSD). Baseline data from Codron et al. (2007), collected from 2002 to 2005 during the dry seasons, are included in (a) for comparison.

**Figure 3:**
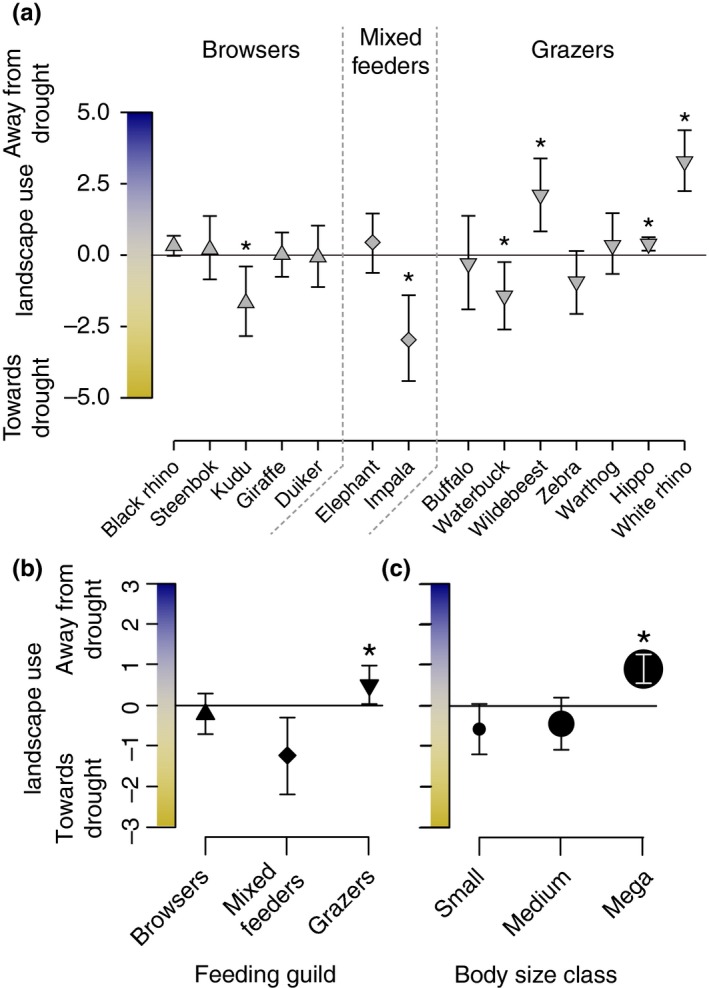
Changes in landscape use by species (a), feeding guild (b), and body size class (c). Change in landscape use is calculated by species as the slope of ∆ dung ~ ∆ rain, where ∆ dung refers to the change in dung density on a log scale and ∆ rain to the proportional change in rainfall between 2016 and 2017. Slopes have been corrected for the overall trend across all species and thus represent relative values. Asterisks (*) denote significant differences (*p* < 0.05). Though included here (a), species‐specific responses are not particularly informative because dung counts can be stochastic at these sample densities (Cromsigt et al., 2008)

Data were analyzed in R 3.5.1 using the package “car” (Fox & Weisberg, 2011), which corrects sums of squares for unbalanced designs (necessary because species are not distributed equally among dietary guilds and body size classes, and because dung sample collection was not balanced either). To examine differences in dietary composition and landscape distributions among species (Figures 2 and 3), we used ANOVAs with diet composition or dung detection intensity as dependent variables and year (as a proxy for drought severity) plus species, guild, or size class as independent variables. Pairwise comparisons were done via Tukey's Honest Significant Difference test (Tukey's HSD). Dung counts were log‐transformed to meet assumptions about normality.

Dietary flexibility might also depend on local drought severity. To address this possibility, we tested whether elephant and impala diet composition changes varied spatially according to drought severity. Dung δ^13^C was averaged within 0.195° × 0.195° raster grid cells (*N* = 26) across the Kruger landscape separately for 2016 and 2017. Values from 2017 were subtracted from 2016 values to generate spatial patterns of diet composition change. These were then modeled with a linear model on local rainfall anomaly. Maps of residuals for both impala and elephant diet composition anomalies showed no evidence of spatial autocorrelation.

To test whether diet change and movement differed by dietary guild and body size class (Figures 2 and 3), we calculated overall changes in diet composition and herbivore landscape use by species from 2016 to 2017. Change in diet composition was calculated, per species, as the overall change in diet C4 fraction from 2016 to 2017. Change in herbivore landscape use was computed, per species, as the slope of the linear response of the change in dung detection intensity from 2016 to 2017 to the proportional reduction in local rainfall for the same period. Formally,

$$\Delta \;\text{landscape use}=\left[\log\left({\text{dung}}_{2016}\right)-\log\left({\text{dung}}_{2017}\right)\right]/\left[\left({\text{rain}}_{2016}-{\text{rain}}_{2017}\right)/{\text{rain}}_{2017}\right]$$

corresponding to movement toward (+) or away (−) from drought refugia as a coefficient with units of log‐transformed herbivore density. To deal with changes in herbivore dung detection arising from changes in grass biomass and dung decomposition rates during drought (Cromsigt et al., 2008), we corrected these slopes for the overall trend in the response across species by subtracting overall coefficients from species‐specific ones (see Table S2 in Supporting Information for slope values), on the assumption that overall herbivore numbers changed relatively little compared with changes in the spatial distributions of herbivores. Coefficients in Figure 3 thus represent relative differences among species. We also modeled changes in dung detection intensity linearly on distance to permanent rivers. We then used either diet composition change or the herbivore landscape use coefficient as the response variable and guild and size class as independent variables in ANOVAs, using Tukey's HSD for post hoc comparisons. As differences in digestive physiology could also underlie differences in strategic responses, we used herbivore landscape use coefficient as the response variable and digestive physiology (ruminant vs. nonruminant) as the independent variable in an ANOVA, as well as in combination with body mass and grass dependence as additional independent variables.

**Figure 4:**
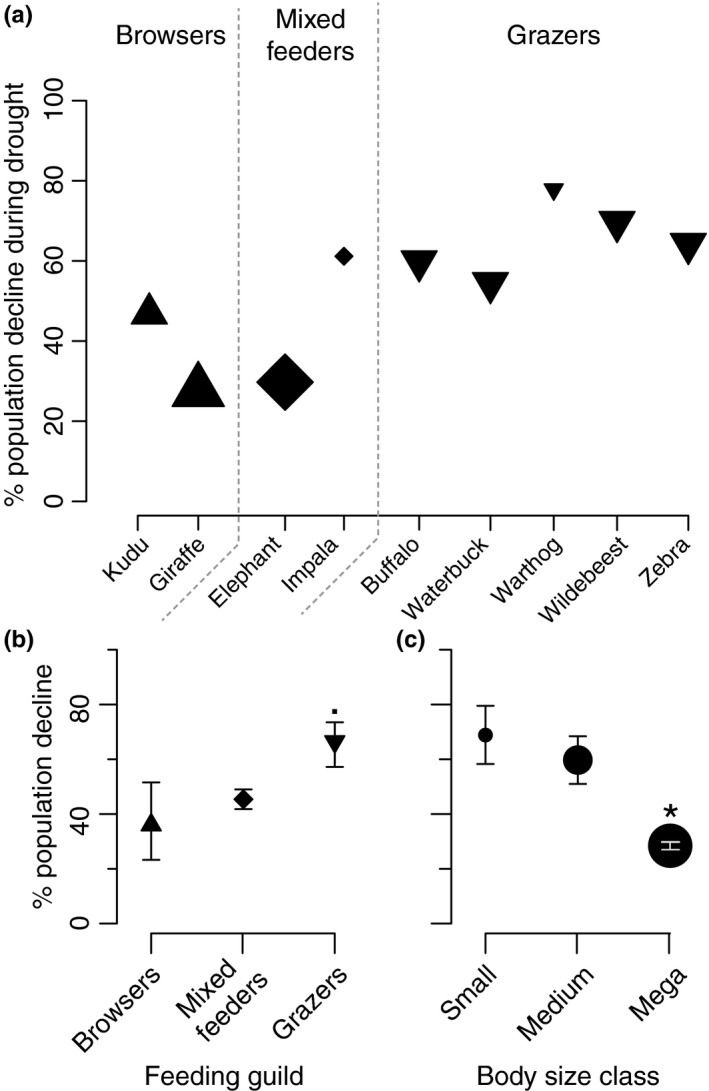
Drought‐induced herbivore population declines by species (a), feeding guild (b), and body size class (c). Population decline estimates are drawn from Young (1994). An asterisk (*) denotes significant difference (*p* < 0.05), and a dot (•) indicates marginal significance (*p* < 0.10). Standard deviations are not included in (a) because sample sizes are too small

Finally, to evaluate how robust strategies might be for mitigating drought‐induced mortality, we used an ANOVA with mortality by species (from Young, 1994; see above) as a dependent variable and feeding guild or body size as independent variables (Figure 4).

## 3 RESULTS

Overall, herbivores differed in their diet composition during and after drought. Herbivores consumed less C4 grass during the drought (*F*_21, 536_ = 111.6, *p* < 0.0001). However, this trend was driven by diet shifts in mixed feeders; elephants (*F*_1,165_ = 36.02, *p* < 0.0001) and impala (*F*_1,150_ = 30.27, *p* < 0.0001), the only mixed feeder species for which dung was collected, significantly changed their diet to incorporate more browse in drought years relative to postdrought years (Table 1, Figure 2). Functional diet breadth of other species, grazers and browsers alike, differed only slightly (and insignificantly) between the two periods (Table 1, Figure 2), well within the range of possible changes in plant δ^13^C from changes in plant water stress (Ehleringer et al., 1993; Farquhar et al., 1989). Shifts in specific species composition likely did occur, though, due to exceptional forage scarcity coupled with changes to vegetative communities during drought (O'Connor, 1995). For impala, dietary shifts were geographically structured. Where drought anomalies were more severe, impala diets shifted significantly more (*F*_1,25_ = 10.28, *p* < 0.005), whereas elephants’ dietary shifts were not significantly related to drought intensity (*F*_1,25_ = 0.063, *p* = 0.8029).

In terms of herbivore landscape use, we counted more dung overall during the drought than after, especially in places where rainfall decreases were more severe (Table S2). Superficially, this might indicate that herbivores had a preference for heavily droughted parts of the landscape, but a more trivial explanation is that dung was more detectable and decomposed more slowly where grass biomass declined more (Cromsigt et al., 2008), or at least that issues with detectability are impossible to rule out in favor of other less trivial mechanisms. Because grass biomass reductions during the drought were widespread despite drought heterogeneity (Staver et al., 2019), dung probably did become more detectable (particularly in portions of the landscape with the largest rainfall anomaly), resulting in the negative overall response of dung count anomaly to rainfall anomaly. Estimated confidence intervals suggest that relationships between dung and rainfall anomalies for browsers and mixed feeders paralleled this overall negative relationship (Figure 3b, Table S2) and therefore cannot be confidently said to reflect any ecological process other than the detectability issues we note above.

By contrast with this overall trend of increasing dung detectability with increasing drought, grazer dung instead decreased where drought was more severe, yielding a positive response of dung anomaly to rainfall anomaly (Figure 3b, Table S2). This positive relationship (despite the above detectability issues) suggests that grazers were unique among feeding guilds in moving substantially to less drought‐affected parts of the landscape (*F*_5,2472_ = 10.83, *p* < 0.0001; Figure 3b), while the other feeding guilds may not have changed their landscape use during the drought.

Herbivore digestive physiology did change whether herbivores moved to drought refugia. Nonruminants altered their landscape use significantly more than ruminants, moving away from the drought (*F*_3,2474_ = 22.58, *p* < 0.0001). However, the effect of digestive physiology was entirely captured by grass dependence and body mass; when all three independent variables were included in an ANOVA, digestive physiology had no effect on how much herbivores moved (*F*_1,2472_ = 0.164, *p* = 0.685), whereas both feeding guild (*F*_2,2472_ = 11.32, *p* < 0.0001) and body size class (*F*_2,2472_ = 8.75, *p* < 0.001) remained highly significant determinants. This suggests that, at least in Kruger, digestive physiology is correlated with body mass and grass dependence, and may not independently influence herbivore drought responses.

Megaherbivores, too, responded to drought by changing their landscape use patterns, on average shifting toward wetter areas, whereas herbivores of smaller body sizes did not (*F*_5,2472_ = 26.79, *p* < 0.0001). As with grazers, megaherbivore dung decreased where drought was more severe, yielding a positive response of dung anomaly to rainfall anomaly despite detectability issues (Figure 3c, Table S2) and indicating that they moved to parts of the landscape less affected by drought. Relationships for small and medium herbivores paralleled the overall negative relationship (Figure 3c, Table S2)—again, perhaps driven by the detectability of dung—suggesting that they did not move significantly during the drought.

Distance from permanent rivers did not significantly affect herbivore landscape use during the drought (*F*_1,2476_ = 2.576, *p* = 0.1086). This was true of all herbivores, subsetted both by feeding guild (*F*_5,2472_ = 9.269, *p* < 0.0001) and size class (*F*_5,2472_ = 23.76, *p* < 0.0001)—though there was marginal support for grazers occurring closer to rivers during the drought (*F*_5,2472_ = 9.269, *p* = 0.0647).

Finally, we found differences among rates of herbivore mortality during drought that paralleled the accessibility of drought mitigation strategies, with differences in mortality depending on body size class and possibly also on feeding guild (*F*_4,4_ = 19.25, *p* < 0.01; Figure 4). Megaherbivores experience significantly less mortality during drought than do smaller herbivores (*F*_2,4_ = 14.98, *p* < 0.05; Figure 4c). Grazers perhaps experience higher mortality during droughts than do either browsers and mixed feeders, though this was only marginally significant, possibly due to small sample size (*F*_2,4_ = 5.26, *p* = 0.076; Figure 4b).

## 4 DISCUSSION

Here, we found (a) that mixed feeders changed their diet during drought to include less C4 grasses, but that drought did not induce dietary shifts in grazers and browsers. However, (b) grazer landscape use shifted toward areas that were less severely impacted by drought, whereas (c) increases in mixed feeder and browser dung counts in severely droughted areas probably reflected dung detectability. Interestingly, (d) large body size did not affect whether herbivores altered their diet, but (e) did increase the probability that herbivores moved toward nondroughted areas. Mortality in droughts paralleled the accessibility of these mitigation strategies, (f) with megaherbivores experiencing significantly lower mortality than smaller (and less mobile) herbivores, and (g) grazers (with the more drought‐affected forage resource and less diet flexibility) experiencing marginally higher mortality than browsers and mixed feeders.

### 4.1 Drought and diet switching

Here, we found that some savanna herbivores shifted to browsing more heavily during drought than normal, but that these shifts were restricted to herbivores with seasonally mixed feeding strategies (see Figure 2). By comparison, browser and grazer diets were broadly similar between drought and normal years, despite evidence that C4 grasses decrease during droughts (Sala et al., 2012), whereas drought‐specialist C3 forbs often increase in abundance during drought (O'Connor, 1995). Slight shifts in diet breadth and composition may have occurred, but were far from significant and well within the range of possible changes in plant isotopic signatures due to water (Ehleringer et al., 1993; Farquhar et al., 1989). That diet flexibility was so prescribed by feeding guild is surprising, because there is little evidence that herbivores in different feeding guilds differ consistently in diet morphology or physiology (Gordon, 2003). Moreover, the result contrasts with results from metabarcoding studies arguing for substantial variation in diet composition across guilds (Kartzinel et al., 2015), although note that metabarcoding work is able to address species‐level diet composition, whereas isotope approaches can only address plant functional diet composition. Nonetheless, whatever the mechanism, herbivore diet breadth during this drought did appear to be constrained to existing diet breadth in normal seasons, at least at the plant functional level.

Observed increases in browsing among impala were larger where drought was more severe, likely reflective of the local availability of trees relative to grasses. By contrast, the browse fraction in elephant dung did not depend on local drought severity. Elephants are more mobile than impala, capable of traveling many kilometers in a single day (Wittemyer et al., 2007), with longer gut passage times due to their size (Clauss et al., 2003). Together, these factors may decouple their diet from the location of their dung, such that their size masks any spatially heterogeneous signal in their responses to drought. Overall, changes in diet did not seem to depend on herbivore body size, but movement did (see Figures 2c and 3c), consistent with a substantial literature showing that large herbivores are more mobile, which we discuss in detail below.

The behavioral responses by mixed feeders to drought may have more general implications for the ecology of savannas during drought. To date, hypotheses about drought impacts on the tree layer have largely focused on direct physiological responses by trees to drought (Fensham, Fairfax, & Ward, 2009) or on the potential implications of grass decreases during drought for release from grass competition or fire (February, Higgins, Bond, & Swemmer, 2013). Overall resilience of browser populations to drought (reflected in relatively low mortality rates despite their lack of drought mitigating behaviors) suggests that trees probably do resist drought effects, at least on short time scales, better than grasses do (Augustine, 2010; Walker et al., 1987). However, the behavioral responses documented here by mixed feeders—an overall dramatic increase in browsing rates during drought, particularly in places that are most affected by drought—likely exacerbate the impacts of drought on the tree layer beyond direct physiological impacts ([Link]). This process merits direct experimental attention.

### 4.2 Drought and movement

While grazers did not change their diets during the drought, they seem to have consistently changed their landscape use more than browsers or mixed feeders, moving toward less drought‐affected areas where grass productivity was higher (Sala et al., 2012). This finding is consistent with work from aerial population censuses (Staver et al., 2019), showing that Kruger's buffalo moved toward the north of the park, where the drought was less extreme. That grazers moved more to avoid drought suggests either that grazers may be more able to travel long distances than other guilds or that drought impacts on vegetation (especially grass) make it more necessary for them to move. Certainly, grazers elsewhere exhibit migratory behavior (Frank et al., 1998; Fryxell & Sinclair, 1988), and locally zebra and wildebeest were historically migratory before Kruger was fenced (Whyte, 1988). Thus, an evolutionary or local ecological history of migration may make it possible for grazers to coopt long‐distance movement as a drought response. However, this does not necessarily imply that they are uniquely able to move substantial distances; instead, grazers may be most heavily impacted by drought effects on vegetation, if grass responds more immediately to drought than trees do (Sala et al., 2012). Higher mortality of grazers suggests that the impacts of droughts on grazers are indeed severe.

Both movement capacity and forage requirements likely contributed to another observation: Megaherbivores changed their landscape use substantially during drought, moving, like grazers, to parts of the landscape less affected by drought. This was consistent with our prediction that larger animals might move more than smaller ones, due to some combination of their ability to move—they are able to travel greater distances (Damuth, 1981; Hein et al., 2012; Owen‐Smith, 1988) and are less constrained by predation (Sinclair et al., 2003)—and their forage requirements—they can eat poor quality food because of their large size but have bulkier requirements (Hopcraft et al., 2010). Interactions between these factors likely account for the significantly lower mortality experienced by megaherbivores during drought.

Notably, movement as a strategy for drought mitigation is only possible in a large reserve such as Kruger and even then only when the park spans major gradients in drought severity. Droughts are sometimes widespread, however, such that migrating away from drought is not possible, a problem that may be aggravated in smaller and fenced reserves (Augustine, 2010). This appears to have been the case during the widespread 1992 drought in Kruger, when buffalo mortality was much more dramatic than it was during this recent drought (Staver et al., 2019). Global warming is expected to increase the spatial extent and severity of droughts (Knapp et al., 2008; Lyon, 2004). Grazers could thus be most vulnerable to these increases in drought severity and homogeneity, experiencing higher rates of drought‐induced mortality, as we have demonstrated here. Habitat fragmentation, particularly from fencing of small reserves, may already have eroded the resilience of grazer populations to droughts (Harris et al., 2009; Wilcove, 2008), and maintaining as much connectivity in landscapes as possible will be crucial to large‐grazer conservation efforts in the face of predicted increases in the frequency and intensity of drought events.

## 5 CONCLUSIONS

**Figure 5:**
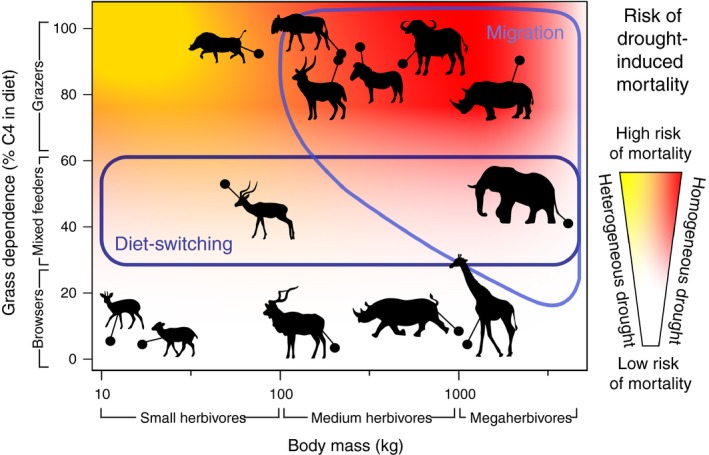
Conceptual synthesis. Grass‐dependent species suffer particular declines in forage availability during droughts. Species with flexible diets (mixed feeders) compensate by incorporating more browse, whereas sufficiently large grazers migrate instead. However, movement may be a less robust strategy than diet switching, because drought refugia are not always available. Background shading reflects herbivore susceptibility to drought‐induced mortality: Strategies expected to result in high mortality during heterogeneous droughts are colored *yellow* and those resulting in mortality only during large and homogeneous droughts *red*; strategies always resistant to mortality are colored *white*. Accessibility of migration as a strategy is outlined in *light blue* and of diet switching in *dark blue*. Diet composition data correspond to grass dependence during a “normal” season (Codron et al., 2007). Body mass data are from Hempson et al. (2015) and mortality from Young (1994)

Overall, our results suggest that herbivores responded to drought by not responding (browsers), by changing their diets (mixed feeders), or by moving to drought refugia (grazers and megaherbivores; Figure 5) and that these shifts are exaggerations of behaviors that they exhibit during normal conditions. Accordingly, mixed feeders responded to drought by amplifying their normal dry season response and incorporating more browse in their diet (Codron et al., 2007), while grazers and some megaherbivores changed their landscape use to access forage reserves, likely ranging over a wider area than in normal years (Riginos, 2015). While the need to migrate seems dependent upon variability of the grass layer (Fryxell & Sinclair, 1988), the capacity to travel long distances appears linked to body size (Hein et al., 2012): This may contribute to the apparent dearth of small‐bodied grazers in more drought‐prone arid savanna systems (Hempson et al., 2015). Browsers, particularly small taxa, did not respond behaviorally to drought and yet simultaneously experience lower mortality (see also Augustine, 2010; Walker et al., 1987). They may survive by avoiding the energetic costs of migration, or trees may simply be more resistant to drought than grasses, thus providing a more dependable source of forage.

From a conservation perspective, it should be reassuring that, in some systems, herbivores can mitigate drought impacts behaviorally, either by altering their diets or by moving to drought refugia. Grazer conservation may present a unique challenge, however, since they can only mitigate drought effects by moving. In this large savanna park, moving to drought refugia did decrease mortality (Staver et al., 2019), but grazers do suffer in large, homogeneous droughts (Augustine, 2010; Staver et al., 2019) and smaller parks elsewhere (Young, 1994). As droughts are becoming increasingly severe and widespread with climate change (Knapp et al., 2008; Lyon, 2004) and as savanna landscapes are increasingly fragmented and fenced (Harris et al., 2009; Wilcove, 2008), grazers may experience intensifying impacts. Modeling work and empirical evidence already suggest that landscape fragmentation and the construction of barriers are partially responsible for the collapses of large‐scale grazer migrations (Harris et al., 2009; Whyte, 1988; Wilcove, 2008). Connectivity in savanna landscapes may be crucial to herbivore—especially grazer—conservation into the future.

## CONFLICT OF INTEREST

None declared.

## AUTHOR CONTRIBUTIONS

JOA collected and analyzed the data; ACS designed the study and contributed to analyses; GPH contributed to design and manuscript development. JOA led the writing with contributions from ACS and GPH.

## Data Availability

Data are available in the South African National Park Data Repository: http://dataknp.sanparks.org/sanparks/metacat/judithk.111976.3/sanparks.
