# Supplementary material for: Drought‐response strategies of savanna herbivores
Source: Ecol Evol. 2019 May 22;9(12):7047–56. doi: 10.1002/ece3.5270 (PMC6662422; doi:10.1002/ece3.5270)
Supplement: Supplementary file 1 [file ECE3-9-7047-s001.docx]

**SUPPORTING INFORMATION**

| **Table S1.** Comparison of dung δ^13^C values among Kruger National Park herbivores between a drought and post-drought year. | | | | | | | | | | | | | | | |
| --- | --- | --- | --- | --- | --- | --- | --- | --- | --- | --- | --- | --- | --- | --- | --- |
| Common name | | Scientific name | Body  mass (kg)* | Dung δ^13^C during drought (2016) | | |  | Dung δ^13^C post-drought (2017) | | |  |  | Baseline dung δ^13^C † | | |
|  |  |  |  | n | Mean | SD |  | n | Mean | SD | *p°* |  | n | Mean | SD |
| Browsers | |  |  |  |  |  |  |  |  |  |  |  |  |  |  |
| Steenbok | *Raphicerus campestris* | 11.2 | 9 | -28.0 | 0.5 |  | 18 | -28.8 | 0.7 | 1 |  | 17 | -26.5 | 2.2 |  |
| Giraffe | *Giraffa camelopardalis* | 1117.5 | 4 | -27.4 | 0.5 |  | 16 | -28.2 | 0.5 | 1 |  | 99 | -26.5 | 0.8 |  |
| Kudu | *Tragelaphus strepsiceros* | 202.3 | 19 | -27.6 | 0.7 |  | 37 | -28.2 | 0.9 | 1 |  | 75 | -26.7 | 0.6 |  |
| Duiker | *Sylvicapra grimmia* | 16.9 | 9 | -26.5 | 1.1 |  | 18 | -28.0 | 2.1 | 1 |  | 8 | -26.5 | 0.9 |  |
| Mixed feeders | |  |  |  |  |  |  |  |  |  |  |  |  |  |  |
| Elephant | *Loxodonta africana* | 4101.8 | 86 | -26.0 | 2.3 |  | 81 | -24.0 | 1.9 | < 0.001 |  | 946 | -25.4 | 3.2 |  |
| Impala | *Aepyceros melampus* | 49.1 | 73 | -23.6 | 1.9 |  | 79 | -21.3 | 3.0 | < 0.001 |  | 325 | -19.6 | 3.0 |  |
| Grazers | |  |  |  |  |  |  |  |  |  |  |  |  |  |  |
| Waterbuck | *Kobus ellipsiprymnus* | 211.8 | 6 | -17.8 | 2.8 |  | 6 | -17.1 | 3.5 | 1 |  | 47 | -14.2 | 1.2 |  |
| Buffalo | *Syncerus caffer* | 486.3 | 15 | -18.3 | 3.1 |  | 10 | -16.0 | 1.6 | 0.43 |  | 176 | -14.7 | 1.0 |  |
| Wildebeest | *Connochaetes taurinus* | 220.1 | 6 | -16.6 | 0.9 |  | 16 | -15.4 | 1.0 | 1 |  | 85 | -14.2 | 1.1 |  |
| Zebra | *Equus burchelli* | 280.4 | 20 | -15.7 | 0.7 |  | 18 | -15.4 | 1.4 | 1 |  | 81 | -14.0 | 1.0 |  |
| Warthog | *Phacochoerus africanus* | 75.9 | 5 | -14.7 | 0.9 |  | 7 | -14.1 | 0.6 | 1 |  | 32 | -14.0 | 1.2 |  |
| *n*, number of samples; SD, ± 1 standard deviation; *p*, *p*-value  * Herbivore body mass data are from Hempson *et al.* 2015.  ° *p-*values correspond to Tukey HSD  † Baseline data from Codron *et al.* 2007 correspond to a non-drought period and are included for the sake of comparison. | | | | | | | | | | | | | | | |

**Table S2.** Raw slopes of dung anomaly versus proportional rainfall anomaly overall, by feeding guild, by body size class, and by species. The mean value of the slopes and the 95% confidence intervals (CI) are provided.

|  |  | Slope of dung anomaly ~ rainfall anomaly | |
| --- | --- | --- | --- |
| Dataset subset | | Mean value | 95% CI |
| Overall | | -0.2912 | [-0.6160,-0.0058] |
| *Guilds:* | |  |  |
| Grazers* | | 0.2155 | [-0.2503, 0.6813] |
| Mixed feeders* | | -1.5408 | [-2.4883, -0.5934] |
| Browsers | | -0.5006 | [-0.9954, -0.0058] |
| *Body size classes:* | |  |  |
| Small herbivores | | -0.8657 | [-1.4860, -0.2455] |
| Medium herbivores | | -0.7321 | [-1.3724, -0.0917] |
| Megaherbivores* | | 0.6094 | [0.2455, 0.9732] |
| *Species:* | |  |  |
|  | Zebra | -1.2467 | [-2.3462, -0.1473] |
|  | Wildebeest* | 1.8146 | [0.54064, 3.0886] |
|  | Waterbuck* | -1.7447 | [-2.9204, -0.5609] |
|  | Warthog | 0.1225 | [-0.9615, 1.2066] |
|  | Buffalo | -0.5618 | [-2.2130, 1.0895] |
|  | Hippo* | 0.1008 | [-0.0937, 0.2953] |
|  | White rhino* | 3.0235 | [1.9565, 4.0906] |
|  | Elephant | 0.1487 | [-0.8869, 1.1843] |
|  | Impala* | -3.2304 | [-4.7398, -1.7210] |
|  | Black rhino | 0.0323 | [-0.30873, 0.3733] |
|  | Duiker | -0.3131 | [-1.3848, 0.7587] |
|  | Steenbok | -0.0421 | [-1.1652, 1.0811] |
|  | Giraffe | -0.2584 | [-1.0014, 0.4846] |
|  | Kudu* | -1.9216 | [-3.1485, -0.6947] |

* 95% confidence interval does not overlap the overall relationship, and thus indicates a significant change in landscape use, either towards (-) or away from (+) drought-affected portions of the landscape

**Literature cited in supporting information:**

Codron, D., Codron, J., Lee-Thorp, J.A., Sponheimer, M., de Ruiter, D., Sealy, J., *et al.* 2007. Diets of Savanna Ungulates From Stable Carbon Isotope Composition of Faeces. *Journal of Zoology* 273(1): 21–29.

Hempson, G.P., Archibald, S., Bond, W.J. 2015. A continent-wide assessment of the form and intensity of large mammal herbivory in Africa. *Science* 350(6264): 1056-1061.
